# Plant Reactome Knowledgebase: empowering plant pathway exploration and OMICS data analysis

**DOI:** 10.1093/nar/gkad1052

**Published:** 2023-11-20

**Authors:** Parul Gupta, Justin Elser, Elizabeth Hooks, Peter D’Eustachio, Pankaj Jaiswal, Sushma Naithani

**Affiliations:** Department of Botany & Plant Pathology, Oregon State University, Corvallis, OR 97331, USA; Department of Botany & Plant Pathology, Oregon State University, Corvallis, OR 97331, USA; Department of Botany & Plant Pathology, Oregon State University, Corvallis, OR 97331, USA; NYU Grossman School of Medicine, New York, NY 10016, USA; Department of Botany & Plant Pathology, Oregon State University, Corvallis, OR 97331, USA; Department of Botany & Plant Pathology, Oregon State University, Corvallis, OR 97331, USA

## Abstract

Plant Reactome (https://plantreactome.gramene.org) is a freely accessible, comprehensive plant pathway knowledgebase. It provides curated reference pathways from rice (*Oryza sativa*) and gene-orthology-based pathway projections to 129 additional species, spanning single-cell photoautotrophs, non-vascular plants, and higher plants, thus encompassing a wide-ranging taxonomic diversity. Currently, Plant Reactome houses a collection of 339 reference pathways, covering metabolic and transport pathways, hormone signaling, genetic regulations of developmental processes, and intricate transcriptional networks that orchestrate a plant's response to abiotic and biotic stimuli. Beyond being a mere repository, Plant Reactome serves as a dynamic data discovery platform. Users can analyze and visualize omics data, such as gene expression, gene-gene interaction, proteome, and metabolome data, all within the rich context of plant pathways. Plant Reactome is dedicated to fostering data interoperability, upholding global data standards, and embracing the tenets of the Findable, Accessible, Interoperable and Re-usable (FAIR) data policy.

## Graphical Abstract

**Graphical Abstract:**
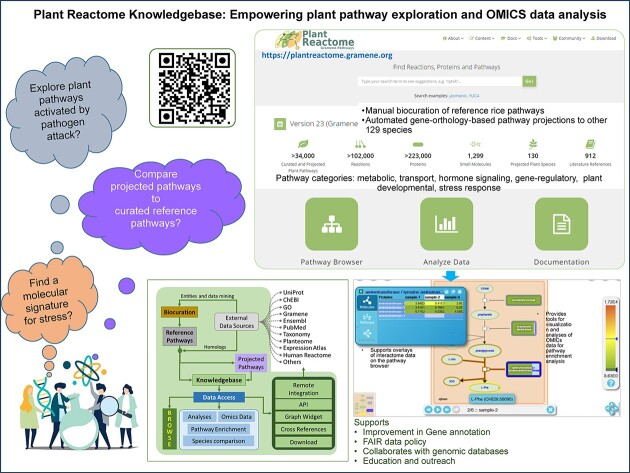

## Introduction

**Figure 1.**
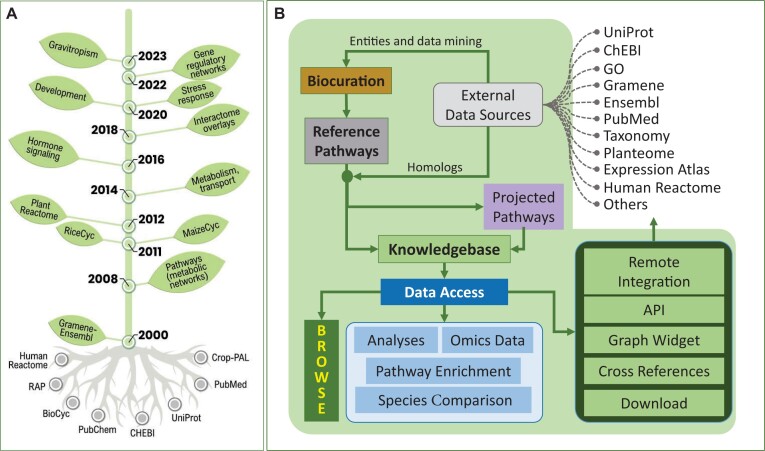
An overview of the Plant Reactome knowledgebase. (**A**) First metabolic networks from RiceCyc (18), previously developed by our group, were lifted and adapted to the Plant Reactome data model for constructing reference metabolic pathways. Subsequently, the pathway model was utilized to represent transport, hormone signaling, plant growth and developmental processes, and transcriptional networks involved in the plant's response to biotic and abiotic stimuli. (**B**) A general schema and pipeline for Plant Reactome production, including cross-referencing with other public databases and resources hosting biological data.

Plant Reactome (https://plantreactome.gramene.org) is an evolving plant pathway knowledgebase. It currently hosts pathways from 130 species ranging from single-cell photoautotrophs to higher plants, including various model organisms, crops, and plants of economic and evolutionary importance (Supplementary Table 1). Since its beginning in 2012, we have continued to develop standard protocols for the biocuration of genes, pathways, and biological processes using the Reactome data model. In addition, we have been extending this data model to reflect plant-specific subcellular structures and other features(1–3). We have expanded our initial curation of metabolic pathways and added processes of hormone signaling, gene regulation, transcription networks, cellular processes like cell cycle, growth and developmental processes, and responses to external stimuli and stresses (Figure 1A). Thus, Plant Reactome includes interactions of small molecules, metabolites, genes, and gene products (proteins, transcripts, miRNA) and connects via cross-references to multiple public repositories and databases (see Figure 1), which host detailed information on genes, gene expression, proteins, metabolites, small molecules, and ontologies (4). For the structural-functional annotations, genes refer to Gramene-Ensembl Plants (5), proteins refer to UniProt (6), and small molecules and metabolites link to ChEBI (7) and PubChem (8,9) (Figure 1). In addition, gene expression data integrated into the Pathway Browser is fetched remotely from EBI-Expression Atlas (10). For molecular interaction data, we partnered with BAR (11) and IntAct (12) and linked to PubMed (13) for literature references. Plant Reactome biocurators utilize Gene Ontology (GO) (14) and Plant Ontology (PO) (15) to describe the gene function(s) and their relationships with plant growth and development (16). EBI-Expression Atlas also lifts the Plant Reactome gene-pathway annotation in bulk. Then, it runs a localized pathway enrichment analysis for each comparative data set or sample within a gene expression experiment (17). Such pathway enrichment annotations are made accessible from their repository.

Plant Reactome provides manually curated pathways for rice (*Oryza sativa*) reference species and gene-orthology-based projections for an additional 129 plant species. The availability of projected pathways to a broad spectrum of plant species and photoautotrophs allows users to compare pathway data across species. Users can select any species to search or browse pathways. Pathway browsing is supported by their hierarchical categorization based on the GO framework of biological processes.

Over the years, we improved the visualization and analysis tools to support pathway comparisons and analysis of omics data uploaded by our users (3,4,19). Our in-built tools allow users to overlay interactome data on the pathway diagram; view basal tissue-specific gene expression data (in the adjacent bottom panel) fetched remotely from Expression Atlas as described in detail previously (3,19); and support navigation to other cross-referenced databases. In addition, users can upload omics data from their projects to conduct pathway enrichment analysis and download the results in graphical or tabular formats. We also encourage Plant Reactome data integration, pathway visualization, and cross-referencing through Application Programming Interfaces (APIs) and other standard formats with other public genomic resources and databases (see Figure 1B). For example, we exchange data, curation practices, and data-formatting protocols with MaizeGDB (20) (https://www.maizegdb.org), AraPort (21) (https://araport.org), Phytozome (22) (https://phytozome-next.jgi.doe.gov), Genome Database for Rosaceae (23) (https://www.rosaceae.org), TreeGenes (24) (https://treegenesdb.org), Legume Information System (25) (https://www.legumeinfo.org), SolGenomics (26) (https://solgenomics.net), PeanutBase (27) (https://www.peanutbase.org), AgBioData consortium (28) (https://www.agbiodata.org), grapevine information system (29) and NASA GeneLab (30) (https://genelab.nasa.gov). In addition, we participate with consortiums, groups, and societies regularly to discuss/formulate/improve on the genomic and pathway data formatting, annotation, biocuration training, and policy issues (28,29,31).

Here, we report the Plant Reactome updates and progress made since our last publication (4) covering Gramene release #62 (November 2019) to the current Gramene release #67 (August 2023). This includes updates in our biocuration strategy, integration of publicly available heterogeneous omics data for synthesizing knowledge about gene networks and pathways, a summary of new and updated pathways, and adding pathway projections for new species. In addition, we summarize our undergraduate biocuration training efforts, community outreach and training, and collaborations with other genomic and pathway databases to promote Findable, Accessible, Interoperable, and Re-usable (FAIR) policy for genomic and pathway data.

## Updates in biocuration strategy

Plant Reactome's data model allows the curation of a wide range of physical entities, including genes (DNA), gene products (e.g. RNA, miRNA, protein), and interactions among them, such as biochemical and regulatory reactions, pathways and processes. We have previously described our adaptation of the Reactome Data Model for the biocuration of reference plant genes and pathways, integration of reference pathways and their gene-orthology-based projection on other species, and public release of the Plant Reactome (4) and integration of plant pathway data to support integrated search in the Gramene (5). The Plant Reactome uses rice (*O. sativa*) as a reference species. We manually curate rice pathways based on information gathered from published experimental studies, functional annotations provided by GO and PO, and additional bioinformatic analysis (e.g. predictions of conserved protein domains, subcellular location, membrane association, etc.). Thus, we use controlled vocabularies for describing pathway entities and events to build interoperability at the level of data annotation. To date, we have integrated information from 912 literature references. When applicable, we link one or more pathways by identifying the preceding reactions to build a system-level network of reactions/pathways.

Living organisms share many housekeeping genes and pathways that exhibit a great degree of functional and structural conservation despite a long history of evolution. Thus, existing knowledge of genes and pathways from the ‘model organisms’ can be leveraged to understand similar processes in other organisms and predict potential genetic and molecular interactions. We also use automated protocols to generate gene-orthology-based pathway projections of reference pathways to other species. This computational approach compensates for the slow pace of manual biocuration and adds tremendous value. Currently (Gramene release #67, August 2023), the Plant Reactome hosts 2129 gene products mapped to 1953 reactions associated with 339 manually curated reference rice pathways and pathway projections for 129 other photoautotrophic species (see Supplementary Table 1). In total, we are hosting >34 000 pathways (manually curated and projected) comprising >102 000 reactions, >223 000 gene products, and 1299 small molecules. Thus, combining two approaches of evidence-based manual biocuration and computational approach for pathway projections helps us to provide pathway knowledge for the broader community of plant researchers and educators.

More recently, we have been focusing on how best to depict plant growth and developmental processes, including organ formation, tissue and cell differentiation, and connect these processes with a plant's response to biotic (e.g. microorganisms, pests, etc.) and abiotic (gravity, light, water, and nutrient availability, change in temperature, day length, etc.) stimuli present in their immediate surroundings (for new and updated pathways see Supplementary Table 2). In general, plant growth and developmental processes are regulated in response to the various signals present in the external environment by discrete actions of different plant hormones (32–34). The plant hormones function synergistically or antagonistically in a complex interactive network that regulates specific aspects of seed germination, organ formation, plant architecture, transition from one developmental stage to another, and eventually senescence and cell death (35–39). Hormone signaling is also vital for biotic and abiotic responses that determine plant development and survival (40–43). Secondly, as plant cells develop, they divide to form various vegetative and reproductive structures. Thus, conserved processes and events associated with the cell division cycle/cell cycle are of crucial importance to plant growth and development. We note here that curated hormone biosynthesis, signaling pathways, and cell cycle pathways in the Plant Reactome provided a base for formulating a curation strategy for plant developmental processes. In the following section, we elaborate on the challenges and strategies used for the biocuration of new types of pathways, including plant organ formation and plant response to extrinsic stresses.

### Plant growth and developmental pathways

In the Plant Reactome, growth and developmental pathways are grouped into two broad categories: vegetative structure development and reproductive structure development. Currently, the vegetative structure development pathways contain two children pathways: (i) regulation of leaf development and (ii) root structure development. Furthermore, we have created sub-pathways to facilitate visualization of several aspects of specific anatomical structure development of plant organs. For example, the Root structure development has separate representations for (i) root hair development, (ii) primary root development, (iii) crown root development, (iv) lateral root development and (v) root elongation. The nesting of related pathways, using parent-child relationships, helps us to link the associated pathways for easy browsing and visualization of pathway data. The reproductive structure development pathways represent the formation of reproductive organs (i.e. Inflorescence development, Anther and pollen development, Seed development, etc.) and the temporal changes during the life cycle of plants that are required for transition from vegetative to reproductive phase (e.g. reproductive meristem phase change, flowering time). We relied on the information available in the published scientific literature for the biocuration of these pathways. Overall, the majority of the reactions associated with the plant developmental pathways represent transcription regulation events consisting of DNA, RNA, proteins, metabolites and small molecules. We have linked plant developmental pathways and reactions with hormone metabolism and signaling pathways wherever applicable. Plant hormones function synergistically or antagonistically, forming a complex interactive network to regulate plant development and organ formation. For example, auxin is involved in regulating the number of branches and panicle (inflorescence) development via several regulatory genes (44–46), and the *LONELY GUY* (*LOG*) gene encodes an enzyme that converts inactive cytokinin into the active form of cytokinin and is required to maintain reproductive shoot meristem activity in rice (47).

We like to note that many genes and molecular interactions that regulate plant growth and development are important determinants of agronomic traits, including seed germination, plant height, plant architecture, stem attributes, flowering times, fertilization, and seed setting (40,48–57). For example, seed size is an important agronomic trait in cereal crops, including rice. The seed is comprised of an embryo and the endosperm. The endosperm occupies most of the space in the seeds of cereal crops and is the major tissue for reserve accumulation. The seed size is a complex quantitative trait controlled by polygenes, miRNAs, and hormone signaling, which influence seed length, weight, thickness and seed filling (Supplementary Figure 1). Thus, to represent various interconnected aspects of seed development, we have manually curated the ‘Seed development’ pathway, including four sub-pathways: (i) regulation of embryo development, (ii) endosperm morphogenesis, (iii) regulatory network of nutrient accumulation and (iv) regulation of seed size. Using this example, we show that the Plant Reactome data model successfully accommodates the complex aspects of plant growth and developmental processes and allows the biocuration of a range of entities, including regulatory proteins and small RNAs.

### Conserved cellular processes

**Figure 2.**
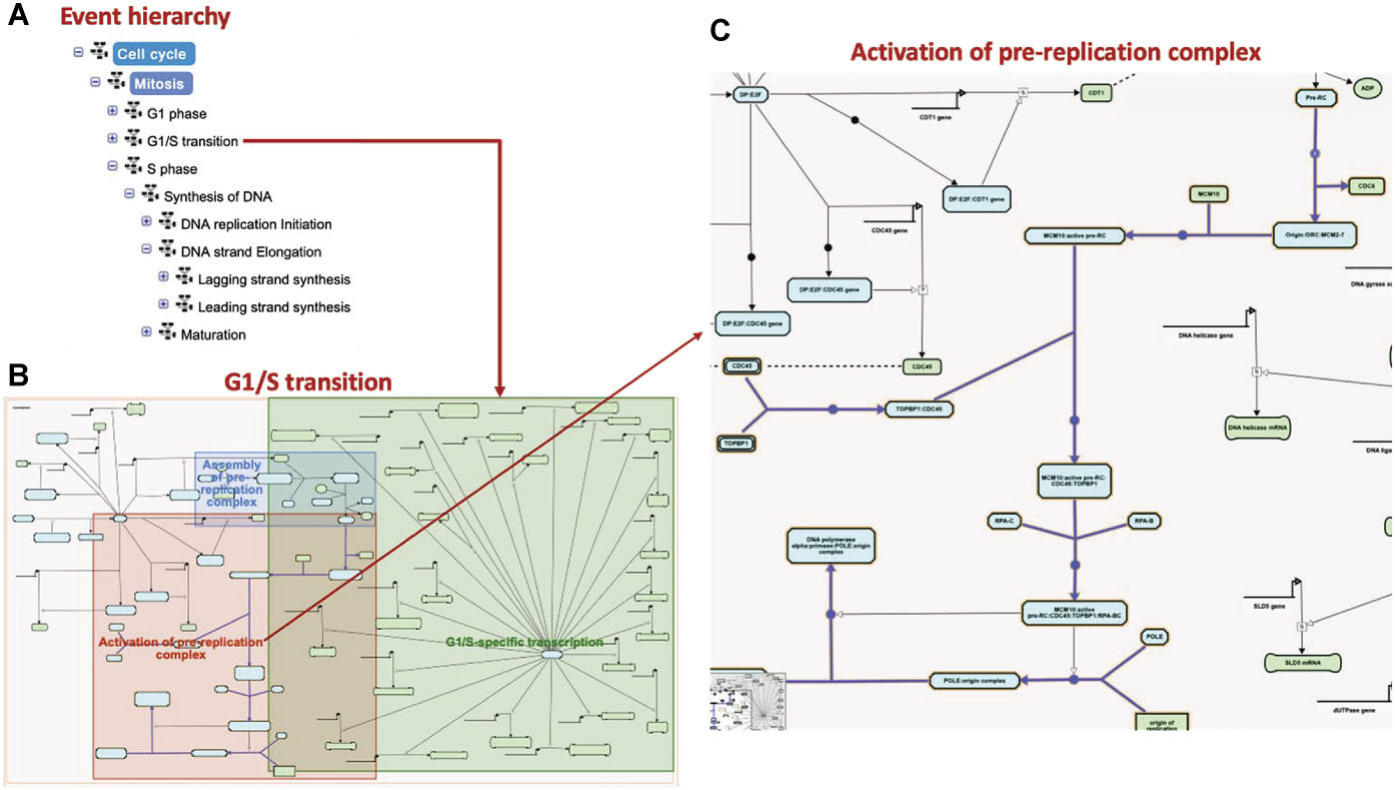
A view of the cell cycle pathway in the Plant Reactome. (**A**) Pathway event hierarchy, (**B**) overview graph of the G1/S transition phase of the mitosis, and (**C**) a zoom-in view of the reaction events of the sub-pathway, activation of pre-replication complex.

We have also biocurated pathways representing conserved cellular processes, such as events associated with the cell cycle. These events are dynamic and represent the transition of cells from one stage to another. For cell cycle pathways, we applied a new strategy for pathway visualization. We have organized the nested pathways to facilitate the visualization of multiple transition sub-pathways within a single browser window. Figure 2 shows various phases of the cell cycle associated with mitosis, which can be viewed in a single pathway diagram, and clicking on the individual child pathway highlights the associated reaction network. We synthesized mitosis associated events based on review of published research papers. We extracted information for 73 proteins, 56 reference genes with associated reference transcripts, and 13 chemical compounds to curate various reaction-like events to represent macromolecular associations, macromolecular transitions, gene-regulations and catalytic reactions.

### Response to abiotic stimuli

We are currently curating gene regulatory networks to depict how biotic and abiotic stimuli/stresses influence specific developmental stages of a plant. For example, we curated a network of transcription factors (TFs) involved in regulating seed germination and coleoptile elongation under submergence (40). Besides reviewing the information in the published literature, we analyzed a previously published transcriptome dataset (58) to identify a TF co-expression network distinctly regulated in response to submergence in tolerant rice genotypes. Our analysis identified 57 TFs that served as nodes for constructing this network. Conceptually, a line connecting two TFs could represent an interaction. We collected empirical evidence from the literature about gene function(s) and gene-gene, gene-protein, and protein-protein interactions to establish the interaction between the transcription factors. In addition, we conducted a thorough analysis of the promoter region of each TF gene and searched for potential TF-target relationships within this group. Finally, a TF network was synthesized by combining independent lines of evidence, including a review of published scientific literature, gene co-expression data, and TF binding sites' presence in the target genes' promoter region (40). Considering that the regulation of gene expression occurs at the transcription level, we used black box reactions to show TF-target relationships between the two TFs in this network. This allowed us (i) to create simple reactions (without showing the details of gene transcription, protein synthesis, and subsequent protein localization in the nucleus— common knowledge) and (ii) to use these reactions as a reference for generating pathway projections for other species (as our pathway projection pipeline uses protein sequence as an input and the projection of DNA/RNA sequences are not supported). Thus, we have extended the information base for synthesizing the reactions and have learned to exploit the backbox reactions for simplifying gene regulatory networks. Moreover, this work revealed several vital interactions between TFs involved in seed germination, organ differentiation, submergence response, and early response to gravitropism. Thus, it suggests that plant development is a dynamic process, and its comprehensive understanding requires integrating heterogeneous data, including information on plant genotype, organ/tissue/cell formation, growth and developmental stage, and phenotype(s).

## Plant Reactome tools for discovery and hypotheses generation

The Plant Reactome analysis tools are accessible from the homepage via the ‘Analyze Data’ icon and the ‘Analysis’ icon in the top right-hand side header of the Pathway Browser. The ‘Species comparison’ tool allows users to compare gene-orthology-based inferred pathways from any species (out of 129 species hosted in the Plant Reactome) with reference rice pathways to generate hypotheses about candidate genes/missing genes/reactions/pathways in the query species. This tool is designed to visually compare individual reference and projected pathways. When a user requests a species comparison, for example, between rice and Arabidopsis, we recommend that the user navigate the pathway hierarchy on the left-hand side panel after submitting a pathway comparison request. This allows one to visualize comparisons at the pathways, sub-pathways, and reaction levels quickly. The comparison results can also be downloaded in a spreadsheet format. Users should not wait for the complete graphic projection of pathway comparisons between two species. The graphic depiction only briefly summarizes how different pathway classes are organized and an overall graphical summary of pathway overlaps, and it takes a few minutes to complete the projection of all pathways. The 'Analyze Data' tool allows uploading and analysis of user-defined omics data (i.e. transcriptomes, metabolomes, proteomes, interactomes) in the context of system-level pathway networks. In our previous publications (3,4), we have described data formatting requirements, data upload process, data visualization and analysis, and accessing/downloading results in graphical and tabular formats.

The Plant Reactome data can be used for developing hypotheses, discovery, and data analytics in many ways. For example, Gupta *et al.* (59) conducted pathway enrichment analysis for transcriptome data to study changes in the gene expression pattern when rice seedlings are shifted from dark to light. This analysis identified 45 light-regulated genes, including those that regulate the mitotic cell cycle pathway events. Using the data file from Gupta *et al.* (Supplementary Table 3), we briefly show how pathway enrichment analysis can be done using the Plant Reactome. Using ‘Analyze Data’ tool, we uploaded a tab-delimited data file containing differential gene expression data from dark- and light-grown rice seedlings and then, using the left-hand-side panel, we investigate the impact of dark to light transition on the expression of genes associated with various pathways. For example, Supplementary Figure 2 shows that the genes associated with the brassinosteroid signaling pathway show down-regulation in light-grown seedlings and support the evidence that photomorphogenesis represses the brassinosteroid signaling (60). A user can explore and select multiple differentially regulated pathways during photomorphogenesis and formulate plausible data-driven hypotheses about candidate genes and pathways where empirical evidence is unavailable. Similar analysis can be done for other transcriptome, proteome, and metabolome datasets.

**Figure 3.**
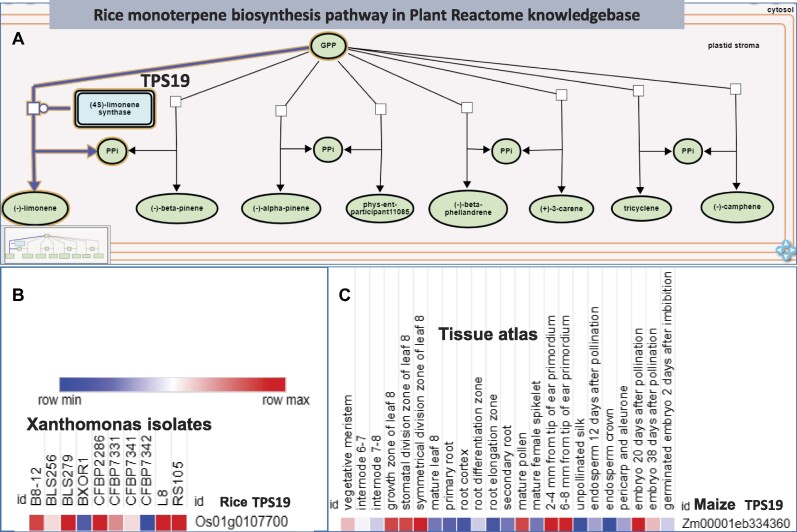
Rice *TPS19* (Os01G0107700) gene mapped in Plant Reactome's pathway network (**A**) and its known expression profile in response to different isolates of *Xanthomonas oryzae* (**B**). The maize homolog Zm00001eb334360 is known to be expressed in various plant tissues.

In addition, researchers can use the Plant Reactome knowledge graphs of gene–gene/gene–protein/protein–protein interactions and their relationships to the repertoire of metabolites, biochemical and small molecules for disease diagnosis, metabolic modeling, and crop yield prediction in multiple scenarios of growth conditions and environments. This can be accomplished by combining Plant Reactome with data and additional analysis tools from outside sources. For example, the arms race between pathogens and plant host species has been waged since before crops evolved into the species they are today. Surviving ancestors long ago evolved numerous resistance strategies to resist pathogen attack. For example, the induction of jasmonate (JA) biosynthesis and signaling pathway in response to pathogen attack is conserved in both dicots and monocots, meaning it evolved before the divergence of these clades 200 million years ago. Therefore, it is logical to consider elevated jasmonate levels as a potential signature of a pathogen infection. In addition to generic signals, like elevation in a phytohormone JA, some specific enzymes and metabolites are regulated in response to pathogen attack. For example, limonene synthase encoded by *TPS19*(Os01g0107700) in rice is a member of a large terpene synthase gene family, whose overexpression leads to improved resistance to blast disease caused by *Magnaporthe oryzae* (Figure 3). The gene was well annotated in the monoterpene biosynthesis pathway in the Plant Reactome. Using the publicly available gene expression data (61) from Expression Atlas (E-GEOD-67588), we found this gene expresses in response to many strains of bacterial pathogen *Xanthomas oryzae pv. oryzicola* (Figure 3B). Many *X. oryzae* strains cause bacterial leaf streak (BLS) disease in rice. Considering the presence of orthologs of *TPS19* in maize (Zm00001eb334360) and its transcription profile, one can hypothesize that limonene synthase is likely to be involved in immunity response in maize. Currently, The *TPS19* gene or encoded enzyme or the reaction product limonene has not been studied for disease resistance in maize, except in a small study reported on seeds infected with Fusarium (62) and reported in an expired patent (63). Though the maize TPS19 ortholog is known to be differentially expressed in many plant structures (64) (E-GEOD-50191) (Figure 3C), however, it remains to be explored if overexpression of maize *TPS19* ortholog in maize provides resistance to any bacterial or fungal pathogen. Limonene has negligible adverse effects on maize growth and development. Thus, it is a worthy research question if limonene can be used as a pesticide to control the onset of certain diseases in maize or other plants. Therefore, the Plant Reactome knowledge graph could help find novel target molecules and genes to formulate approaches that can support early detection of disease signatures or improve disease resistance by identifying novel molecules, candidate genes, and pathways.

Thus, when extended to completeness with enriched data sources and data types, the Plant Reactome knowledge graph will provide high-quality information helping score molecular signatures needed to differentiate and identify disease detection.

## Plant reactome updates

In the current Gramene release #67, August 2023, the Plant Reactome hosts 2129 gene products mapped to 1953 reactions associated with 339 manually curated reference rice pathways. The curated rice genes and pathways were used to generate gene-orthology-based pathway projections for 129 other photoautotrophic species covering a broad taxonomic range (for a complete list, see Supplementary Table 1). Since our last publication (4), 41 new and 19 revised reference pathways were added. Overall, during the manual biocuration of pathways, we have improved the functional annotation of approximately 600 protein-coding genes, including members of two large gene families (50,51).

## Plant Reactome data access

As part of the FAIR data sharing standards, full downloads of Plant Reactome data are available at https://plantreactome.gramene.org/download/currrent. The file gk_current.sql.gz is a compressed dump of the MySQL database, while reactome.graphdb.gz is a compressed dump of the Neo4J database. Other files include text files describing mappings for ChEBI, NCBI, UniProt, Ensembl and miRBase to Plant Reactome pathways and reactions. Images of all pathways and reactions are also available in PNG and SVG formats. For advanced users and integration in third-party resources, programmatic access via RESTful API to JSON data is available via the Content Service (https://plantreactome.gramene.org/ContentService). Examples of usage of the API are available on that page, for example, getting a list of the species represented by executing the command line (curl -X GET ‘https://plantreactome.gramene.org/ContentService/data/species/main’ -H ‘accept: application/json’). All results from the Content Service are returned in JSON formatted responses. Projects using the Content Service to retrieve Plant Reactome data include our partners, Ensembl Plants, Gramene, PubChem, etc. External sites can embed Plant Reactome pathway diagrams by implementing our DiagramJS widget. Instructions are available at https://plantreactome.gramene.org/index.php?option=com_content&view=article&id=58 to add the javascript code to any website. The initial diagram can be set within the options and items to highlight, such as genes or reactions.

## Outreach and training

We delivered 15 presentations at conferences, including the Biology of Genomes (2020 virtual); 4th International Conference on Plant Synthetic Biology, Bioengineering, and Biotechnology (2020 virtual); 2nd Session of the 14th Annual Biocuration Conference (2021 virtual); AgBioData consortium's monthly webinar series in 2022; NASA GeneLab's Analysis Working Group Symposium (2022 virtual); Plant and Animal Genome (PAG 30) conference, 2023. Training plant researchers and students in biocuration is crucial (65). In the past three years, we have trained 14 undergraduate students in the biocuration of genes and pathways. In the process, they learned to critically review scientific literature, and developed skills needed for knowledge synthesis by utilizing information from diverse sources (see biocuration of genes and pathways videos from a few students at https://www.youtube.com/playlist?list=Pliue2y1LJ89BjJBcHy6dmaLaNq9s0t2iA). We continue to update functional annotations of genes and gene families during manual biocuration of pathways, share these updates with other appropriate public databases, and disseminate this information in published articles (50,51). We regularly participated in monthly meetings organized by the AgBioData consortium and NASA Plant Analysis Working Group (Plant AWG). Our team members have contributed to a white paper on genotype and phenotype data standardization (31). We have also contributed to a meta-analysis of plant biology data to study microgravity and space environment (66), and a decadal survey for 2023–2032, Thriving in Space (67). We have also contributed to GO discussions (14) and addressed the current state of tools and resources for pan-genome data (68).

## Conclusion and future directions

In the era of multi-omics data, Plant Reactome provides evidence-based curated knowledge graphs of macromolecular interactions and gene-networks associated with plant metabolic, genetic, developmental, and stress response processes. For biocuration of genes and pathways, we rely primarily on the published scientific literature and use the Evidence and Conclusion Ontology (https://www.ebi.ac.uk/ols/ontologies/eco) to describe types of scientific evidence (i.e., laboratory experiment, computational prediction, author statement, etc.) to support a reaction/pathways/molecular interactions. More recently, we have been analyzing the transcriptome data available in the public domain for improving gene functional annotations, associating genes with biological processes and pathways, and extracting gene co-expression networks (40,50,51). In addition, we are using data on transcription binding sites to infer potential TF–target relationships. For example, we have synthesized a transcription factor network involved in seed germination under submergence in rice by combining more than one line of evidence (40). In another study, Clark *et al.* (69) have described an approach for integrating transcriptomics and phospho-proteomics data to infer gene regulatory networks. We intend to exploit publicly available multi-omics data to extend the biocuration of gene regulatory networks using similar approaches.

In another study, Thessen *et al.* (16) recently identified Arabidopsis and Sorghum homologs involved in the drought response using a knowledge graph approach supported by multi-omics data. We expect to leverage the current data structure to integrate information on plant diseases, host-pathogen interactions, abiotic stress response, phenotypes, and traits of agronomic importance. In the near future, we also look forward to the application of Language Learning Models and OntoGPT frameworks (70,71) to discover novel interactions and patterns associated with genotype-environment-phenotype (GxE = P), besides supporting the biocuration of pathways (40,50).

As we transition from the genomic to the pan-genomic era, new opportunities and challenges emerge. Making connections between the genetic components, environment, and the resulting phenotype is of tremendous value for improving the crop yield and survival of plant species in the face of ongoing global climate change. Pathway databases provide an excellent platform to integrate data and knowledge gained from heterogeneous sources using appropriate ontology frameworks and organize data in the broader context of the system-level networks of pathways and biomolecular interactions (72–78). Since Plant Reactome pathway data is curated at the species level, it can easily accommodate the knowledge gained from pan-genome analysis. Indeed, data from the pan-genome will help to fill the gaps (pathway holes) where critical genetic hotspots or genomic loci metabolism are absent in the reference genome. Thus, pan-genome data can help to build/predict a relatively accurate species-specific or clade-specific metabolic network (68). Likewise, the new omics data generated from single cells can be analyzed using the Plant Reactome and help researchers define the unique and shared features (proteins, pathways, and metabolites) between different plant cell types. We expect that Plant Reactome will continue to meet the demands of our users, evolve to accommodate new data types, and bring innovation in data analyses and visualization.

## Supplementary Material

gkad1052_Supplemental_Files

## Data Availability

The data and resources discussed above are available from the Download page, accessible from the main navigation bar at https://plantreactome.gramene.org.
