# Supplementary material for: Plant Reactome Knowledgebase: empowering plant pathway exploration and OMICS data analysis
Source: Nucleic Acids Res. 2023 Nov 20;52(D1):D1538–47. doi: 10.1093/nar/gkad1052 (PMC10767815; doi:10.1093/nar/gkad1052)
Supplement: gkad1052_Supplemental_Files [file gkad1052_supplemental_files.zip › Supplementary Table 2.docx]

**Supplementary Table 2.** A summary of new and updated contents in the Plant Reactome knowledgebase

| **Biocuration of Genes and Pathways** | | | | **New species** | |
| --- | --- | --- | --- | --- | --- |
| Release# 62 (November 2019) | | | | | |
| -Pathway category: cellular processes   - Mitosis   - G1 phase   - G1/S Transition   - Assembly of pre-replication complex   - Activation of pre-replication complex   - G1/S-specific transcription   -Pathway category: metabolism   - Allantoin degradation - Amino propanol biosynthesis - Ascorbate biosynthesis - Vitamin E biosynthesis - Arginine biosynthesis - Asparagine biosynthesis - Beta alanine biosynthesis I - Beta alanine biosynthesis III - Tyrosine degradation I - Ammonia assimilation cycle - Ureide biosynthesis | | | | - Dicots   - *Cannabis sativa*   - *Cannabis sativa subsp. Indica*   - *Capsella rubella*   - *Citrullus lanatus*   - *Corchorus olitorius*   - *Cynara cardunculus var. Scolymus*   - *Humulus lupulus*   - *Humulus lupulus var. lupulus*   - *Nelumbo nucifera*   - *Salvia hispanica* - Monocots   - *Asparagus officinalis*   - *Eragrostis tef*   - *Phyllostachys edulis*   - *Zoysia japonica* - Embryophyte   - *Marchantia polymorpha* | |
| Release# 63 (October 2020): | | | | | |
| -Pathway category: cellular processes:   - DNA replication   -Pathway category: plant development   - Primary Root elongation, - Crown root initiation, emergence, and development - Lateral root initiation, emergence and development   -Revised pathways categories: cellular processes and metabolism   - Primary root development - Flower development - Floral bract development - Photorespiration - Cardiolipin biosynthesis - UDP-L-arabinose biosynthesis and transport - Vitamin E biosynthesis - Ascorbate biosynthesis | | | | - Dicots   - *Citrus clementina*   - *Ipomoea triloba*   - *Olea europaea var. Sylvestris*   - *Pistacia vera*   - *Prunus avium* - Monocots   - *Ananas comosus*   - *Eragrostis curvula*   - *Saccharum spontaneum* - Charophyceae   - *Chara brauni*i | |
| Release# 64 (September 2021): no updates | | | | | |
| 270 Gene-orthology based pathway projections were added for Zea mays ver5 | | | | | |
| Release# 65 (April 2022) | | | | | |
| 2435 gene-orthology based pathway projections for 12 new species were added. | | | - Dicots   - *Camelina sativa*   - *Chenopodium quinoa*   - *Cucumis melo*   - *Ficus carica*   - *Juglans regia*   - *Lactuca sativa*   - *Olea europaea*   - *Rosa chinensis*   - *Sesamum indicum* - Monocots   - *Setaria viridis* - Ranunculales   - *Papaver somniferum* - Nymphaeaceae   - *Nymphaea colorata* | | |
| Release# 66 (December 2022): no updates | | | | | |
| Release# 67 (August 2023) | | | | | |
| -Pathway category: gravitropism, plant development   - Regulation of seed germination and coleoptile growth under submergence and normal gravity environment - Regulation of lemma joints development and leaf angle by cytokinin | | | | - Dicot   - *Brassica juncea*   - *Corymbia citriodora*   - *Pisum sativum*   - *Quercus lobata*   - *Quercus suber*   - *Vigna unguiculata* - Monocot   - *Digitaria exilis*   - *Echinochloa crus-gallis*   - *Lolium perenne*   - *Secale cereale* | |
