# Supplementary figures and images for: Plant Reactome Knowledgebase: empowering plant pathway exploration and OMICS data analysis
Source: Nucleic Acids Res. 2023 Nov 20;52(D1):D1538–47. doi: 10.1093/nar/gkad1052 (PMC10767815; doi:10.1093/nar/gkad1052)

# miRNA-mediated transcription regulation

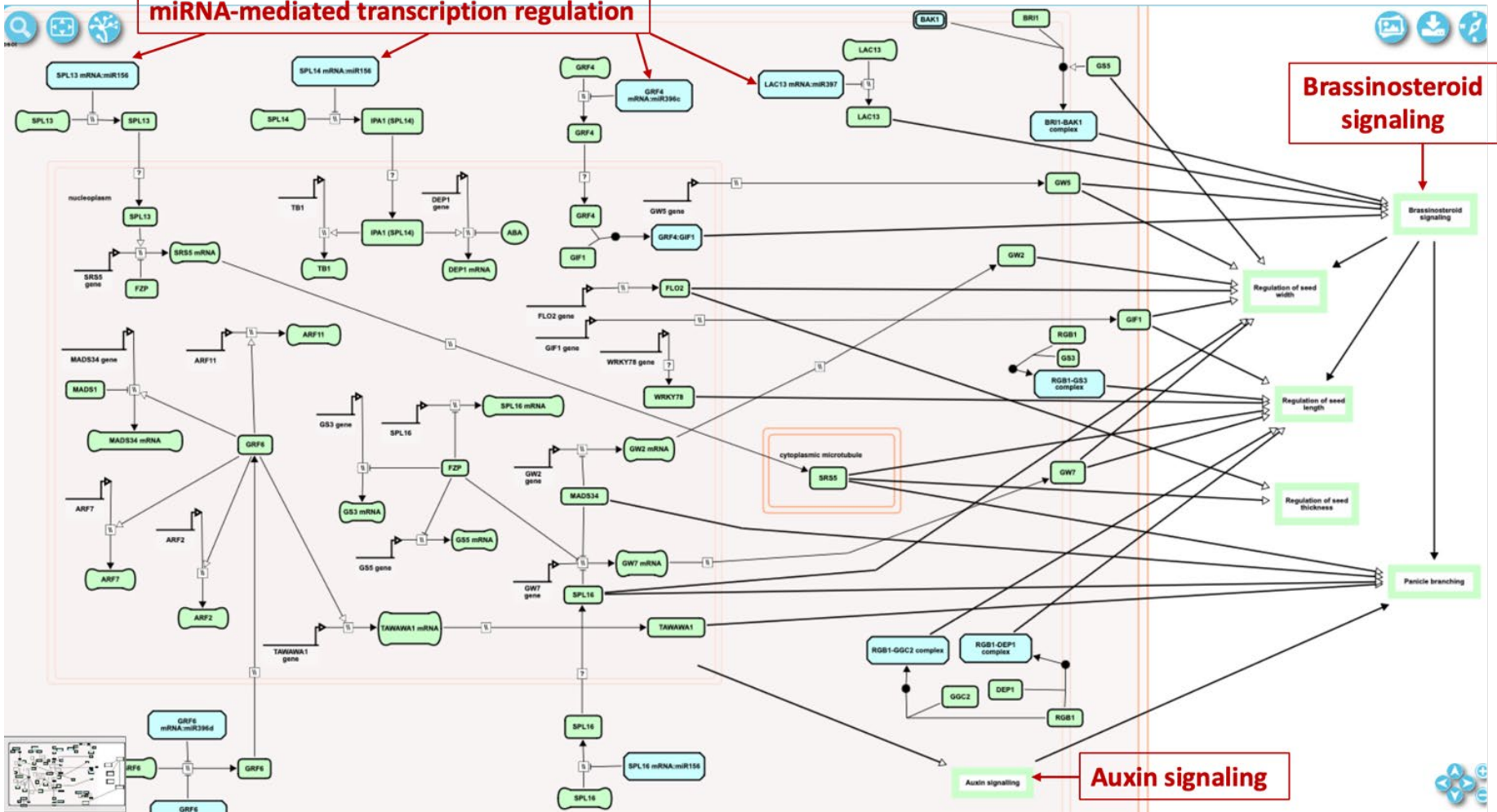

Supplement: gkad1052_Supplemental_Files [file gkad1052_supplemental_files.zip › Supplementary Figure 1.pdf]
