# Supplementary material for: Plant Reactome Knowledgebase: empowering plant pathway exploration and OMICS data analysis
Source: Nucleic Acids Res. 2023 Nov 20;52(D1):D1538–47. doi: 10.1093/nar/gkad1052 (PMC10767815; doi:10.1093/nar/gkad1052)
Supplement: gkad1052_Supplemental_Files [file Supplmentary Figure 2.pdf › Supplmentary Figure 2.pdf]

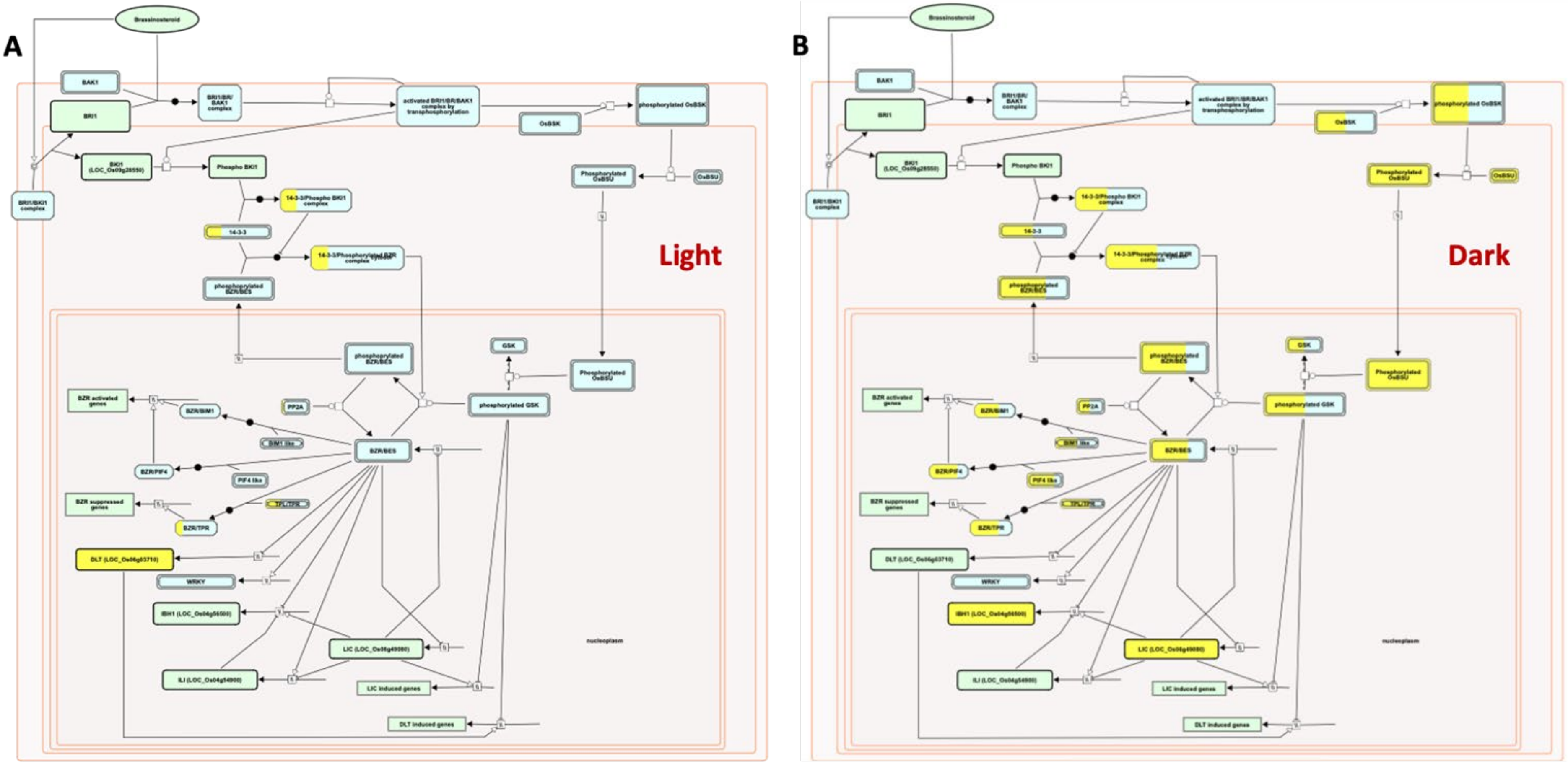

Brassinosteroid signaling
